# Trends in Well-Child Visits With Out-of-Pocket Costs in the US Before and After the Affordable Care Act

**DOI:** 10.1001/jamanetworkopen.2021.1248

**Published:** 2021-03-12

**Authors:** Paul R. Shafer, Alex Hoagland, Heather E. Hsu

**Affiliations:** 1 Department of Health Law, Policy, and Management, School of Public Health, Boston University, Boston, Massachusetts; 2 Department of Economics, College of Arts and Sciences, Boston University, Boston, Massachusetts; 3 Department of Pediatrics, School of Medicine, Boston University, Boston, Massachusetts

## Abstract

This cross-sectional study uses national claims data to assess trends in well-child care visits with out-of-pocket costs before and after passage of the Affordable Care Act.

## Introduction

In the US, both Medicaid and the Children’s Health Insurance Program exclude well-child care from cost sharing, but out-of-pocket costs present a barrier to accessing preventive services for privately insured children.^1^ The promised elimination of these costs is a popular provision of the Affordable Care Act (ACA). Although the proportion of well-child visits with out-of-pocket costs declined from 73% before passage of the ACA to 49% in 2011 and 2012,^2^ the evolution of trends in out-of-pocket costs is unknown. We used national claims data to describe cross-sectional trends in well-child visits with out-of-pocket costs from 2006 through 2018.

## Methods

This cross-sectional study was deemed exempt from review, and the requirement for patient written informed consent was waived by the Boston University Institutional Review Board because deidentified data were used. We followed the Strengthening the Reporting of Observational Studies in Epidemiology (STROBE) reporting guideline. We used health insurance claims from 2006 through 2018 from children aged 0 to 17 years with full-year coverage each year; claims were obtained from the IBM MarketScan Commercial Claims and Encounters Database.^3^

We focused on 2 outcomes: the proportion of children who had an office or outpatient visit without a wellness visit and the proportion of wellness visits resulting in an out-of-pocket cost, which were calculated annually during the study period. We stratified the sample by 2 age groups (0 to 5 years and 6 to 17 years) because these groups have a different recommended frequency of visits for wellness and other preventive services.^4^ Diagnosis codes from the *International Classification of Diseases, Ninth Revision *(visits before October 2015) and *International Statistical Classification of Diseases and Related Health Problems, Tenth Revision* (visits in October 2015 and after) and *Current Procedural Terminology* and Healthcare Common Procedure Coding System codes used to identify preventive services were obtained from the Centers for Disease Control and Prevention and were supplemented with coding guidelines from major insurers.^5^

We examined trends in visit volumes to ensure that compositional changes did not explain the findings and assessed the delivery of preventive services during non-wellness visits. We plotted the trends over time and tested for significance using linear regression. *P* < .05 was considered to be statistically significant, all *P* values were 2-sided. Data were analyzed from June 10, 2020, to January 15, 2021, using SAS, version 9.4 (SAS Institute, Inc) and Stata, version 16 (StataCorp).

## Results

The sample consists of 88 863 727 person-years from privately insured children in 48 states, with a total of 371 573 184 visits across the study period from 2006 through 2018 (Table). The mean (SD) age of participants was 9.19 (5.09) years, and 15 945 616 of 31 247 534 participants were male (51.03%). The proportion of children with at least 1 office or outpatient visit and without a wellness visit declined from 39.3% in 2006 to 29.0% by 2018 (coefficient on linear time trend: −0.79 percentage points; 95% CI, −1.11 to −0.47; *P* < .001) (Figure, A). The volume and relative share of total visits per child (coefficient on linear time trend: 0.01 visits; 95% CI, 0.01-0.02; *P* = .03) and wellness visits per child (coefficient on linear time trend: 0.02 visits; 95% CI, 0.01-0.02; *P* < .001) remained stable over time (Figure, B). Older children had office visits or outpatient care without a wellness visit at higher rates than younger children during the study period (Figure, A). The percentage of wellness visits with an associated out-of-pocket cost declined from 54.2% in 2010 (the year that the ACA was passed) to 14.5% in 2018 (coefficient on linear time trend: −5.63 percentage points; 95% CI −6.96 to −4.31; *P* < .001) (Figure, C). In addition, the percentage of non-wellness visits with associated preventive services increased approximately 60%, from 1.8% in 2006 to 3.7% in 2018 (coefficient on linear time trend: 0.09 percentage points; 95% CI, 0.03-0.15; *P* = .005).

**Table.** Sample Characteristics

Characteristic	Year		
	2006	2018	2006-2018
Age, % (95% CI)			
0-5 y	27.59 (27.55-27.63)	28.71 (28.66-28.75)	27.94 (27.93-27.95)
6-17 y	72.41 (72.37-72.45)	71.29 (71.25-71.34)	72.06 (72.05-72.07)
Sex, % (95% CI)			
Male	51.07 (51.04-51.11)	50.99 (50.96-51.03)	51.03 (51.02-51.04)
Female	48.93 (48.89-48.96)	49.01 (48.97-49.04)	48.97 (48.96-48.98)
Children treated in office or outpatient setting, % (95% CI)			
Had any office or outpatient visit	69.63 (69.60-69.67)	72.52 (72.47-72.56)	71.57 (71.56-71.58)
Had a wellness visit	37.02 (36.64-37.42)	56.41 (56.35-56.45)	48.19 (48.18-48.20)
Children, total No.	5 887 673	4 448 580	31 247 534
Visits, total No.	21 451 976	19 979 162	371 573 184

**Figure.**
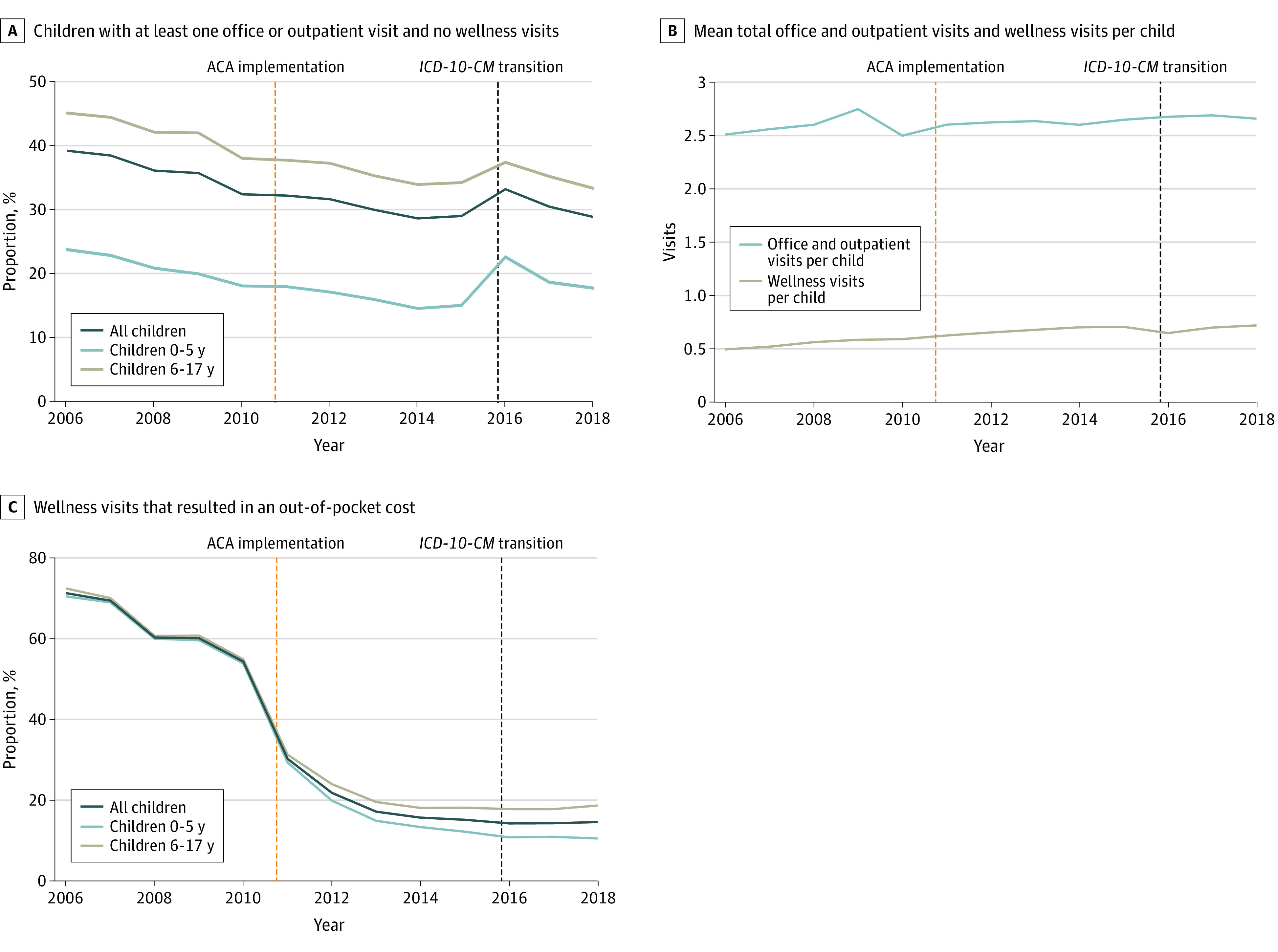
Trends in Pediatric Office and Outpatient Visits and Wellness Visits With Out-of-Pocket Costs, 2006 to 2018 Out-of-pocket costs included costs associated with procedures (eg, laboratory tests, immunizations) that occurred on the same day as the wellness visit in addition to any charges for the visit itself. ACA indicates Affordable Care Act; *ICD-10-CM*, *International Statistical Classification of Diseases, Tenth Revision, Clinical Modification*.

## Discussion

Following passage of the ACA, engagement of privately insured children in well-child care increased and the proportion of families incurring out-of-pocket costs for this care declined. However, approximately 1 of 7 wellness visits still results in out-of-pocket costs. Delivery of preventive services is increasing during non-wellness visits, indicating that providers may be encouraging prevention at any opportunity. This study is limited because specific insurers were not analyzed; however, there is considerable overlap in preventive coding guidelines, and we believe that our coding scheme is inclusive of federal guidance and several major insurers. There are several reasons why parents still receive unexpected bills for well-child care but the continued decline in costs as a barrier is encouraging.^6^
